# Upregulation of miR-335-3p by NF-κB Transcriptional Regulation Contributes to the Induction of Pulmonary Arterial Hypertension via APJ during Hypoxia

**DOI:** 10.7150/ijbs.34517

**Published:** 2020-01-01

**Authors:** Junming Fan, Xiaofang Fan, Hui Guang, Xiaoqiong Shan, Qiuyun Tian, Fukun Zhang, Ran Chen, Fangzhou Ye, Hui Quan, Haizeng Zhang, Lu Ding, Zhuohui Gan, Feng Xue, Yongyu Wang, Sunzhong Mao, Lianggang Hu, Yongsheng Gong

**Affiliations:** Institute of Hypoxia Medicine, School of Basic Medical Sciences, Wenzhou Medical University, Wenzhou, Zhejiang, 325035, China.

**Keywords:** NF-κB, Pulmonary arterial hypertension, miR-335-3p, APJ, Hypoxia

## Abstract

Pulmonary arterial hypertension (PAH) is a cardiopulmonary disease that can lead to heart failure and eventually death. MicroRNAs (miRs) play essential roles during PAH progression; however, their exact mechanism of action remains unclear. Apelin is a small bioactive peptide with a key protective function in the pathogenesis of PAH mediated by binding to the APJ gene. The aim of the present study was to investigate the role of miR-335-3p in chronic normobaric hypoxia (CNH)-induced PAH in mice and the potential underlying regulatory mechanism. Adult male C57BL/6 mice were exposed to normoxia (~21% O_2_) or CNH (~10% O_2_, 23 h/d) for 5 weeks. MiR-335-3p was significantly increased in lung tissue of CNH-induced PAH mice. Blocking miR-335-3p attenuated CNH-induced PAH and alleviated pulmonary vascular remodeling. Bioinformatics analysis and luciferase reporter assay indicated that nuclear factor-kappa beta (NF-κB) acted as a transcriptional regulator upstream of miR-335-3p. Pyrrolidine dithiocarbamate treatment reversed the CNH-induced increase in miR-335-3p expression and diminished CNH-induced PAH. Moreover, p50^-/-^ mice were resistant to CNH-induced PAH. Finally, APJ was identified as a direct targeting gene downstream of miR-335-3p, and pharmacological activation of APJ by its ligand apelin-13 reduced CNH-induced PAH and improved pulmonary vascular remodeling. Our results indicate that NF-κB-mediated transcriptional upregulation of miR-335-3p contributes to the inhibition of APJ and induction of PAH during hypoxia; hence, miR-335-3p could be a potential therapeutic target for hypoxic PAH.

## Introduction

Pulmonary arterial hypertension (PAH) is a devastating lung disease characterized by increased pulmonary vascular resistance, which can lead to right heart failure and even death 1, 2. Structural and functional changes in pulmonary arterial smooth muscle cells (PASMCs) responsible for pulmonary vascular remodeling are thought to represent the main cellular mechanism underlying the development of PAH 3-5. However, the pathogenesis of PAH has yet to be fully delineated.

MicroRNAs (miRs), 20-26 nucleotide-long single-stranded non-coding RNA molecules, participate mainly in the inhibition of post-transcriptional gene expression by interacting with the 3′-untranslated regions (3′-UTR) of their downstream target mRNAs 6, 7. A growing number of studies have shown that miRs are involved in diverse key biological processes including cancer, inflammatory responses, and cellular function 8, 9. Recently, miRs have been strongly associated with development and progression of PAH and the modulation of its pathophysiological function 10, 11. Moreover, they exhibit essential roles in both proliferation and migration of PASMCs, as well as vascular remodeling in PAH during hypoxia, as have been reported by a number of elegant studies 12-22. Although miRs are known major upstream regulators of many key target genes involved in hypoxic PAH development, the transcriptional regulation of miRs by critical effectors on PAH during hypoxia is less clear. Therefore, it is important to uncover how miR activation is associated with other well-defined transcriptional factors in the progression of hypoxic PAH.

The expression of miRs in response to environmental stimuli is often subjected to regulation by transcriptional factors including nuclear factor-kappa beta (NF-κB) 23-26. NF-κB plays an essential role in the modulation of PAH 27-32, and NF-κB activation is one of the primary hypoxia-driven signals to promote pulmonary arterial obliteration, inflammation, and reduced immune regulation in the context of severe obliterative PAH 33. Although miRs are becoming increasingly recognized as important regulators of PAH, comparatively little is known about the specific role of the NF-κB pathway in miR regulation during PAH pathogenesis. These facts led us to hypothesize that NF-κB-mediated regulation of miRs might contribute to the modulation of PAH during hypoxia.

In this study, we first screened and addressed the potential role of miR-335-3p in CNH-induced PAH in mice. We then treated mice with an NF-κB inhibitor, pyrrolidine dithiocarbamate (PDTC), and utilized NF-κB knockout (p50^-/-^) mice to test whether the essential role of NF-κB in CNH-induced PAH depended on upregulation of miR-335-3p. Finally, we investigated whether NF-κB-mediated transcriptional upregulation of miR-335-3p contributed to the inhibition of APJ, a downstream target of miR-335-3p, during the development of CNH-induced PAH.

## Materials and methods

### Animals

Adult male C57BL/6 mice aged 10-12 weeks were purchased from the Animal Center of Wenzhou Medical University (Wenzhou, Zhejiang, China). The animals were housed in groups of four in standard cages (30×20×12 cm), maintained at ambient temperature (22±1°C) and humidity (55±5%), and were kept on a 12-hour light/dark cycle (lights on at 07:00-19:00 hours) with *ad libitum* access to mouse chow and water. The animals were allowed to acclimatize in the animal facility for 1 week before experimental manipulation. All efforts were made to minimize animal suffering.

### Chronic normobaric hypoxia (CNH) exposure

Mice were randomly divided into Normoxia control and CNH groups (N=5-8 per group). For CNH exposure, mice were placed carefully in a normobaric hypoxic chamber with a fraction of inspired oxygen (FIO_2_) of ~0.1, 23 h per day, for five weeks. Mice in Normoxia group were kept in a normobaric chamber at sea level with FIO_2_ of ~0.21, as we described previously 34. Cages were cleaned once daily between 10:00 and 11:00 h.

### MiR-335-3p antagomir treatment in CNH-induced PAH in mice model

To investigate whether there is a preventive effect of miR-335-3p on CNH-induced PAH, mice were randomly divided into four groups (N=5-8 each group): 1) Normoxia+miR-335-3p scramble control, 2) Normoxia+miR-335-3p antagomir, 3) CNH+miR-335-3p scramble control, 4) CNH+miR-335-3p antagomir. MiR-335-3p antagomir or miR-335-3p scramble control were injected intravenously (tail vein, 5 nmol at 0.1 ml) at day 0, 7, 14, 21, and 28, and the mice were sacrificed at day 35.

To test whether there is a therapeutic effect of miR-335-3p on CNH-established PAH model, mice were exposed to CNH for 5 weeks to induce PAH, followed by housing at normoxia condition for remaining 10 weeks. Therapeutic experiment with miR-335-3p antagomir administration was undertaken at 11, 12, 13, and 14 weeks, and the animals were sacrificed at 15 weeks. MiR-335-3p antagomir was synthesized by Ribobio Co., Ltd. (Guangzhou, China).

### Pyrrolidine dithiocarbamate (PDTC) treatment

Mice were randomly divided into four groups (N=5-8 per group): 1) Normoxia+vehicle; 2) Normoxia+PDTC; 3) CNH+vehicle; and 4) CNH+PDTC. Mice in the Normoxia+PDTC and CNH+PDTC groups were subcutaneously injection of PDTC (50 mg.kg^-1^day^-1^), twice daily (10:00 and 18:00 h), and those in the Normoxia+vehicle and CNH+vehicle groups were subcutaneously injected with the same volume of vehicle as PDTC treatment, and exposed to normoxia or CNH treatment, as described above. PDTC was freshly dissolved in normal saline each day before injection.

### Apelin-13 treatment

Mice were randomly divided into four groups (N=5-8 per group): 1) Normoxia+vehicle, 2) Normoxia+apelin-13, 3) CNH+vehicle, 4) CNH+apelin-13. Mice in Normoxia+apelin-13 and CNH+apelin-13 groups were intraperitoneal injection with apelin-13 (15 ng/mice/day), and mice in Normoxia+vehicle and CNH+vehicle groups were intraperitoneal injection with the same volume of vehicle as apelin-13 treatment, and exposed to normoxia or CNH treatment, as described above. Apelin-13 was freshly prepared in normal saline (pH 7.4) each day before injection (10:00 h).

### Measurements of RVSP

The degree of PAH was recorded by measuring right ventricular systolic pressure (RVSP) with right heart catheterization as we previously described 12. In brief, the animals were anesthetized by intraperitoneal injection with pentobarbital (30 mg/kg), ventilated through a transtracheal catheter, and laid in a supine position on a heating platform. A microcatheter was inserted gently through jugular vein into right ventricle (RV) and then pulmonary artery. After an equilibration period for 30 minutes, RVSP was recorded on a physiological recorder (PowerLab) via a transducer (PowerLab 8 passages electrophysiolograph; ADI) connected to the micro-catheter.

### Assessment of right ventricular hypertrophy (RVH)

After RVSP measurement, the animals were sacrificed and hearts were collected. Atrium was trimmed and the free wall of RV was separated from the left ventricle and septum (LV+S). RV and LV+S of each heart were weighted, and the ratio of RV/(LV+S) was calculated to assess RVH. The animals were sacrificed and the lung tissues were harvested and stored at -80 °C until further analysis.

### Morphometric analyses of pulmonary vascular remodeling

To evaluate pulmonary arterial muscularization, lungs of mice were infused and fixed with 4% paraformaldehyde and embedded in paraffin, and lung sections (5 μm) were prepared and stained with Masson trichrome stain as we previously described 35. Distal small arteries with an external diameter between 50 μm and 100 μm for each section were captured using Nikon eclipse biological microscopy (Nikon, Japan) under high magnification. Arterial diameter and area were assessed using Nikon NIS-Elements basic research software for quantitative analyses, and vessel wall area/total cross area was calculated. Morphometric analysis was performed by the observer who was blinded to the experimental treatments.

### Microarray processing and analysis

Microarray processing and analysis for miRs was performed by the Ribobio Co., Ltd. (Guangzhou, China). Briefly, total RNA was extracted using TRIzol reagent (Invitrogen). RNA quality and quantity were measured using a NanoDrop spectrophotometer (ND-1000, NanoDrop Technologies), and RNA integrity was determined using gel electrophoresis. RNA was then reverse-transcribed using the TruSeq RNA Library Prep Kit (Illumina, CA, USA). The libraries were subjected to quality validation using the Agilent 2200 TapeStation, and then paired-end sequenced using HiSeq 2500 (Illumina). The resulting reads were mapped to the rn5 database using TopHat2 36. Genes with an expression change >1.5-fold were clustered and shown in a heat map (log2 scale) using NetWalker62.

### Quantitative real-time polymerase chain reaction (qRT-PCR)

Total RNA was isolated from mice lung tissues using TRIzol reagent (Invitrogen) and was reversely transcribed into single stranded complementary DNA with PrimeScript RT reagent Kit (Qiagen) following the manufacturer's instructions. Amplification and quantification were carried out with SYBR Premix Ex Taq II (Qiagen), and were processed using Applied Biosystems 7500 Real-time PCR System. The primers for pri-miR-335-3p and miR-335-3p and endogenous control RNU6B (U6) were synthesized by Ribobio Co., Ltd. (Guangzhou, China). All samples were amplified in triplicate. The relative mRNA expression levels were quantified using the comparative cycle threshold (CT) method (2^-ΔΔCT^) and expressed as the fold-change compared with a control.

### Western blotting

Lung tissues were lysed in RIPA buffer (1% Triton X-100, 0.5% sodium deoxycholate, 0.2% SDS, 150 mM NaCl, 10 mM Hepes, pH 7.3, 2 mM EDTA, and protease inhibitor mixture; Pierce), as we previously described 12. After blocking, membranes were probed overnight at 4°C with the primary antibodies as follows: APJ (SC-33823; Santa Crutz, USA), Bax (14796; CST, USA), Caspase 3 (9662; CST, USA), Cleaved caspase 3 (9661; CST, USA), ɑ-SMA (14968; CST, USA), CD31 (3528; CST, USA), and GAPDH (2118; CST, USA). HRP-conjugated anti-rabbit or anti-mouse (CST) was used to detect the primary antibodies. After washing, the protein bands were visualized using an enhanced chemiluminescence (Bio-Rad). The relative expression of the proteins was quantified using densitometric scanning and analyzed by the Imaging J System and expressed as percent of controls. Molecular mass was determined relative to protein markers (BioRad).

### Promoter and 3′-UTR luciferase reporter assays

To investigate the transcriptional regulation of NF-κB on miR-335-3p, we constructed a wild-type luciferase reporter vector containing 2260 bp of the mouse pre-miR-335-3p 5′ proximal genomic region and a mutant luciferase reporter vector in which the NF-κB binding sites were deleted. In order to validate the binding of candidate miRs to the APJ mRNA, TargetScan algorithm (http://www.targetscan.org) was applied to predict downstream targets of miR-335-3p and the corresponding binding sites. APJ 3′-UTR was PCR amplified from mice genomic DNA with primer 5′-GCG GCT CGA GCG ATG AAG GAC TAG GGT GAA C-3′ (forward) and 5′- AAT GCG GCC GCC AGA GCC CTC CAA GAA CAA A-3′ (reverse), and inserted into pmirGLO dual-luciferase vector (Promega). To construct mutated 3′-UTR report vector, residues in the region that base-pairs with miR-335-3p seeding sequences were mutated by site-directed mutagenesis. The primers used were listed as follows: APJ-mut, 5′-ACA GGA TGT ACT TTT AAG GGT GAG CTT TTG TGA-3′ (forward) and 5′-TTT TTC ATA AAA GTA GGC AAG AAA GTG GCC TC-3′ (reverse). For luciferase assays, 5×10^6^ HEK293T cells were co-transfected with either APJ reporter plasmids together with miR-335-3p agomir or scramble control, or p65 cDNA and the luciferase reporter constructs described above. Transfection was performed using Lipofectamine 2000 (Invitrogen, ThermoFisher Scientific) according to the manufacturer's instructions. Twenty-four hours after transfection, cells were harvested and luciferase activity assay was measured using the Dual-Luciferase Assay System according to the manufacturer's instructions (Promega), and each well had three replicates. Firefly luciferase activity was normalized to renilla luciferase activity.

### Statistical analysis

Values are presented as the mean±standard error of means (s.e.m). All data were analyzed with One-way or Two-way ANOVA followed by a Tukey's post hoc test using the GraphPad Prism software. Student's t-test was applied for comparisons between two groups. Pearson's correlation coefficients was used to analyze the correlation between miR-335-3p and RVSP, and miR-335-3p and RV/(LV+S). Results were considered to indicate statistical significance at *p*<0.05.

## Results

### CNH exposure induces PAH and upregulates miR-335-3p expression in mouse lung tissue

**Figure 1:**
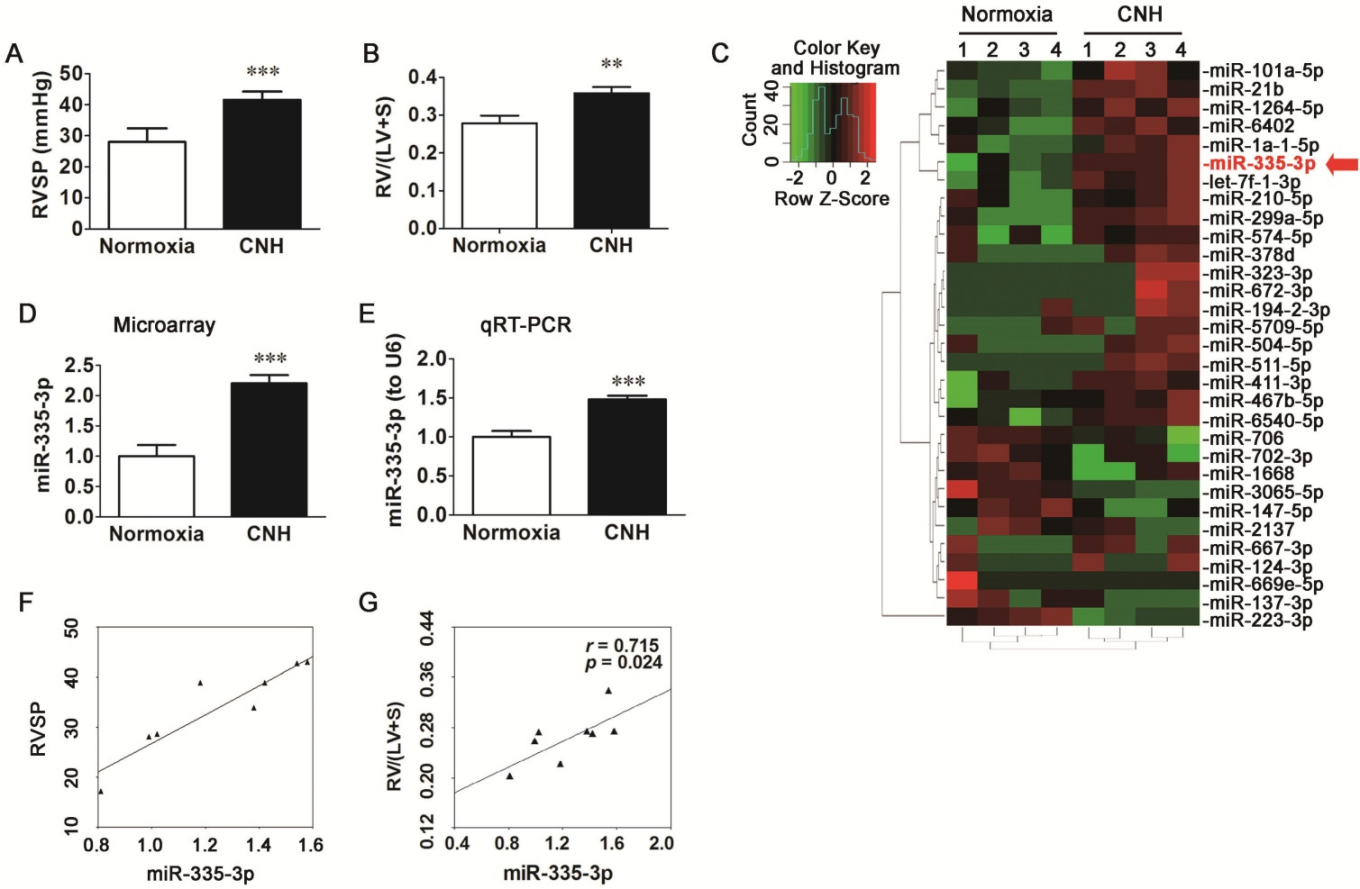
** Chronic normobaric hypoxia (CNH) exposure induces pulmonary arterial hypertension (PAH) and upregulates miR-335-3p expression in the lungs of mice.** CNH treatment significantly increased RVSP **(A)** and RV/(LV+S) **(B)** (N=5-8 per group). **(C)** Heatmap and sample clustering analysis of the differential expressed miRs in the lungs of mice (N=4 per group). Each row represents a miR and each column represents a sample. **(D)** Microarray and **(E)** quantitative RT-PCR analysis of miR-335-3p expression in the lungs of mice. **(F)** Correlation analysis between miR-335-3p and RVSP, and **(G)** Correlation analysis between miR-335-3p and RV/(LV+S) (N=4 per group). Values shown are means±s.e.m. **p<0.01, ***p<0.001 *vs.* Normoxia.

To assess whether CNH exposure could induce PAH in mice, we measured right ventricular systolic pressure (RVSP) and right ventricular hypertrophy (RVH). Mice exposed to CNH had a 48.2% higher RVSP (*p*<0.01) relative to normoxia control animals (Figure 1A) and a 28.3% higher right ventricle (RV)/left ventricle plus septum (LV+S) ratio (*p*<0.01) (Figure 1B).

To determine the role of miRs in the pathogenesis of CNH-induced PAH, we performed a microarray analysis on RNAs isolated from the lungs of mice that were exposed to CNH or normoxia (controls). The overall profiles of the differently expressed miRs are presented as a heatmap (Figure 1C). As shown in Figure 1D, miR-335-3p expression was enhanced in the lungs of mice exposed to CNH compared with those of normoxia controls. The microarray data were confirmed by mature miR-specific quantitative PCR analysis, which revealed a significant increase in miR-335-3p expression in the lungs of mice exposed to CNH (Figure 1E). Pearson correlation analysis revealed that the expression of miR-335-3p in mouse lungs analyzed by quantitative real-time PCR (qRT-PCR) correlated positively with RVSP (Figure 1F, *r*=0.92, *p*=0.002) and RV/(LV+S) (Figure 1G, *r*=0.715, *p*=0.024). We also established a reproducible CNH-induced PAH rat model (FiO_2_ ~10%, 23 h per day, for 2 weeks), as shown in Figure S1. Results of microarray and qRT-PCR analysis revealed that CNH exposure enhanced miR-335 expression in rat lungs (D-F in Figure S1), and that the latter correlated positively with mPAP (G in Figure S1, *r*=0.861, *p*=0.033) and RV/(LV+S) (H in Figure S1, *r*=0.986, *p*=0.001). Together, these results indicate that CNH treatment induces PAH and right heart dysfunction as well as marked changes in miR-335-3p expression in mouse lungs.

### Blocking miR-335-3p abrogates the development of CNH-induced PAH in mice

**Figure 2:**
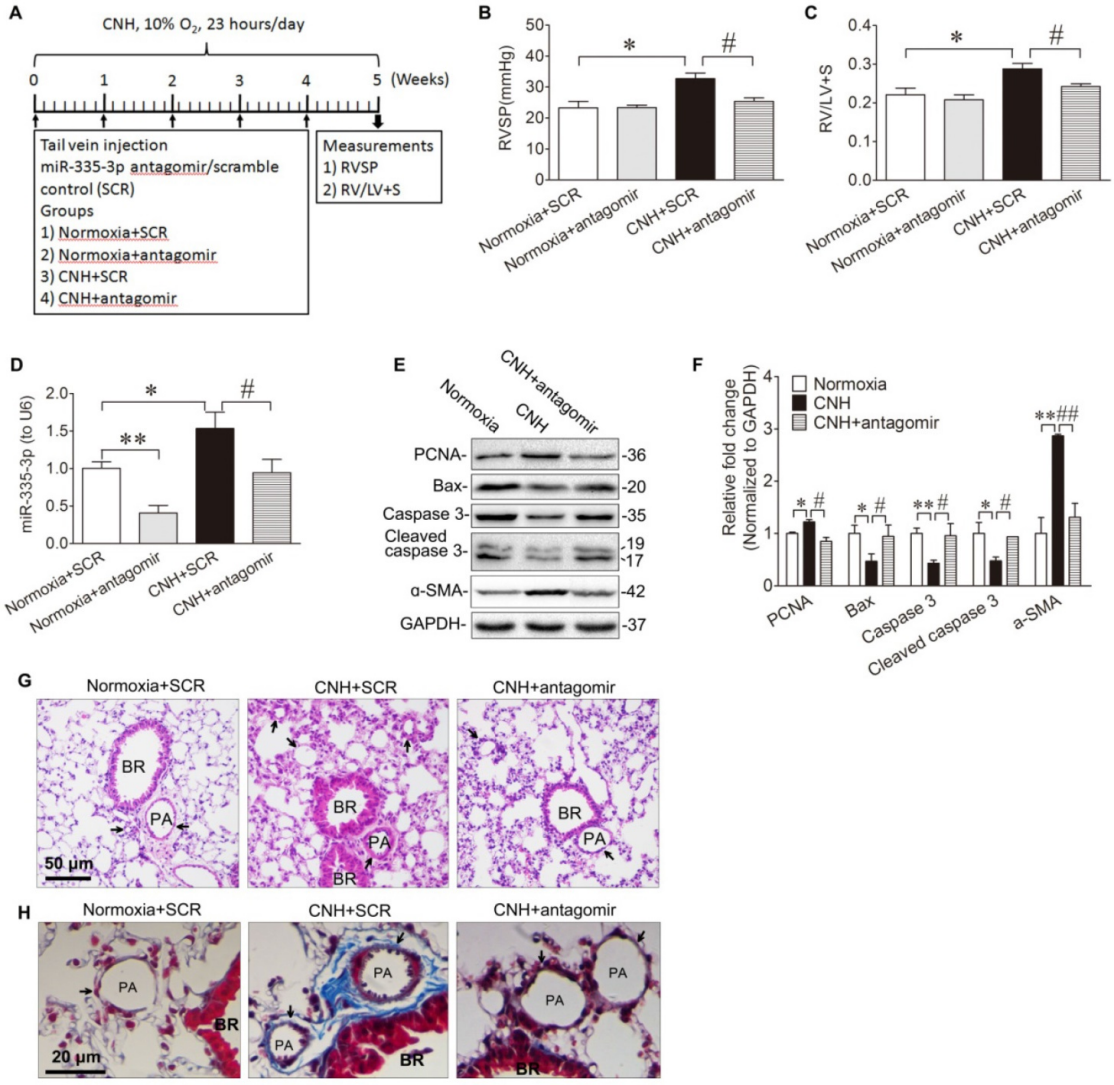
** Blockade of miR-335-3p prevents the development of CNH-induced PAH and pulmonary vascular remodeling in mice. (A)** Schematic diagram of the experimental protocol. MiR-335-3p antagomir treatment reversed CNH-induced increase of RVSP **(B)** and RV/LV+S **(C)**. **(D)** qRT-PCR analysis of miR-335-3p expression in the lungs of mice after treatment of miR-335-3p antagomir. **(E)** Western blotting analysis of relative proteins expression in the lungs of mice with Normoxia, CNH, or CNH+antagomir treatment. **(F)** Quantification of proteins expression bands in (E) was done by densitometry and normalized to GAPDH. **(G)** Representative images of H&E staining of lung sections. **(H)** Representative masson trichrome staining of lung section. Values shown are means±s.e.m. N=5-8 per group. *p<0.05, **p<0.01 *vs.* Normoxia. #*p*<0.05 , ##*p*<0.01 *vs.* CNH+SCR. SCR, scramble control. PA, pulmonary artery. BR, bronchiole.

Given that miR-335-3p expression was increased in the lungs of CNH-induced PAH mice (Figure 1D, Figure 1E), we tested whether blocking miR-335-3p could exert a protective effect during CNH-induced PAH in mice. To this end, we administered 50μl (5μM concentration) of miR-335-3p antagomir (or scrambled control) at day-0, day-7, day-14, day-21, and day-28 after CNH exposure, and the mice were kept in CNH for total 5 weeks (Fig. 2A). Two-way ANOVA indicated that there was a significantly different effect on RVSP between CNH (F_(1,13)_=10.544; *p*=0.009) and CNH with antagomir treatment (F_(1,13)_=4.457; *p*=0.049), while the antagomir alone had no significant effect (F_(1,13)_=4.194; *p*=0.068). A significant difference was observed also regarding the effect of CNH (F_(1,16)_=11.402; *p*=0.005), antagomir (F_(1,16)_=9.728; *p*=0.008), and CNH×antagomir (F_(1,16)_=4.672; *p*=0.05) on the RV/(LV+S) ratio. Post hoc analysis indicated that CNH significantly increased RVSP from 23.2±2.1 to 32.7±1.8 mmHg and RVH index from 0.22±0.02 to 0.29±0.01. In contrast, preventive administration of miR-335-3p antagomir significantly decreased RVSP from 32.7±1.8 to 25.4±1.1 mmHg and RVH index from 0.29±0.01 to 0.24±0.02 (all *p*<0.05) (Figure 2B, Figure 2C). The results of qRT-PCR showed that the endogenous miR-335-3p levels in lung tissues were significantly reduced when antagomir was administrated, as shown in Figure 2D.

Next, we evaluated the role of miR-335-3p in the regulation of CNH-induced cellular proliferation and apoptosis in the lungs. We observed that CNH caused a modest increase in expression levels of proliferating cell nuclear antigen (PCNA) and the PASMCs marker alpha smooth muscle actin (ɑ-SMA). It also caused a reduction in apoptosis manifested by a significant decrease in total caspase 3, cleaved caspase 3, and Bax expression levels. CNH-induced PASMCs proliferation and apoptosis were reversed after the administration of miR-335-3p antagomir (Figure 2E, Figure 2F). This finding suggests that miR-335-3p stimulates lung PASMCs proliferation while inhibiting their apoptosis following CNH exposure *in vivo*. Histological analysis by hematoxylin and eosin and Masson trichrome staining of lung sections showed that, in comparison to the scrambled group, CNH significantly increased vascular remodeling, whereas miR-335-3p antagomir significantly prevented CNH-induced pulmonary vascular remodeling in mice (Figure 2G, Figure 2H).

### Blocking miR-335-3p attenuates established hypoxic PAH in mice

**Figure 3:**
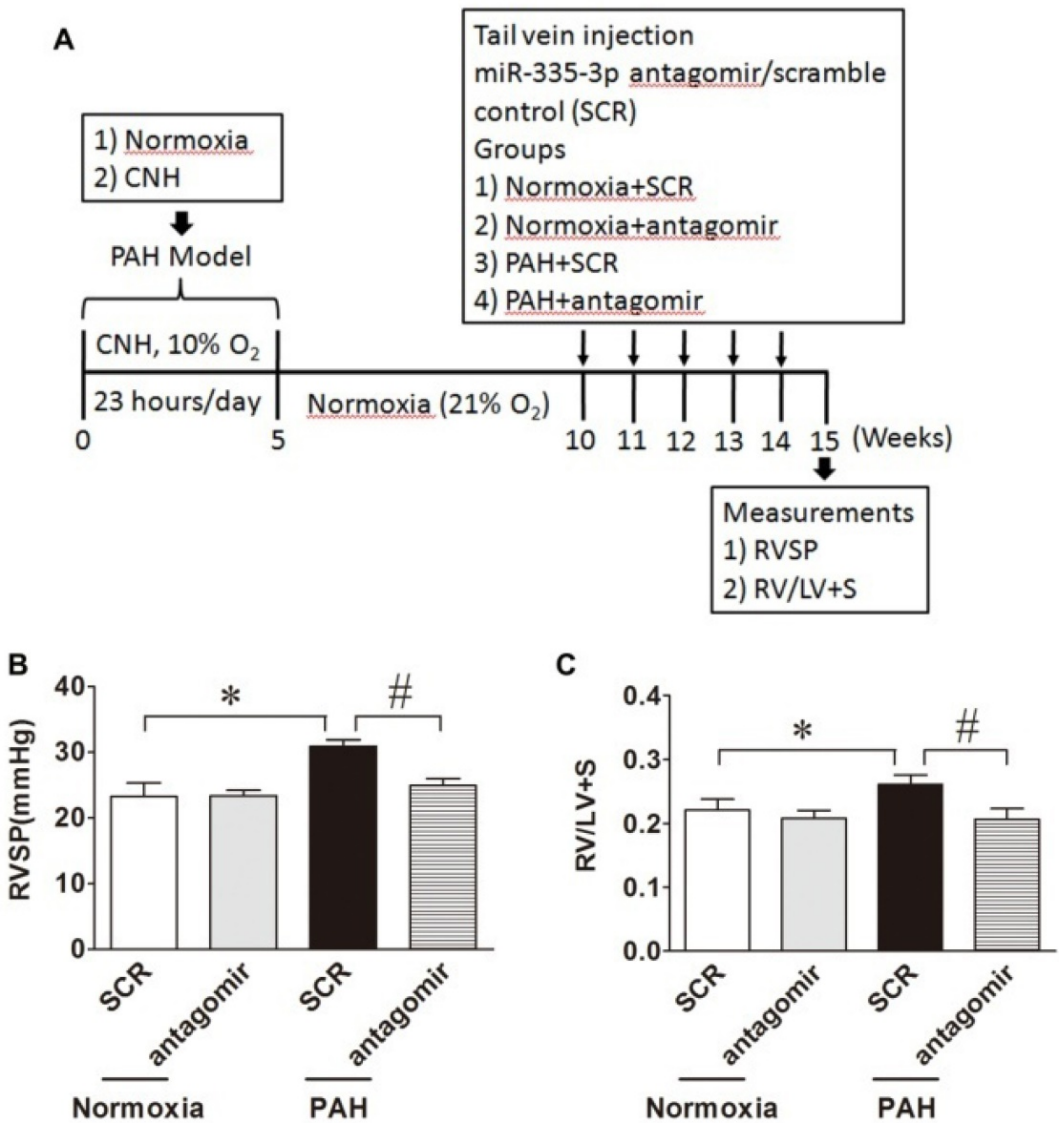
** Blockade of miR-335-3p attenuates established hypoxic PAH in mice. (A)** Schematic diagram of the experimental protocol. MiR-335-3p antagomir treatment attenuates established hypoxic PAH in mice by reversing CNH-induced increase in RVSP **(B)** and RV/LV+S** (C)**. Values shown are means±s.e.m. *p<0.05 *vs.* Normoxia. #*p*<0.05 *vs.* CNH+SCR. N=5-8 per group. SCR, scramble control.

Data presented above (Figure 2) provide evidence that inhibition of miR-335-3p abrogates CNH-induced PAH in mice. We next investigated whether blocking miR-335-3p could attenuate the progression of established hypoxic PAH in mice. To this end, mice were exposed to CNH for 5 weeks followed by a further 5 weeks under normoxic conditions prior to miR-335-3p antagomir (or scrambled control) administration at 11, 12, 13, and 14 weeks (Figure 3A). Results showed that CNH treatment for 5 weeks significantly increased RVSP from 23.3±2.1 to 30.9±0.9 mmHg and RVH index from 0.22±0.02 to 0.26±0.01; whereas miR-335-3p antagomir treatment significantly reduced the increased RVSP from 30.9±0.9 to 24.9±1.1 mmHg and RVH index from 0.26±0.01 to 0.21±0.02 (all *p*<0.05; N=5-8 per group) (Figure 3B, Figure 3C). These findings indicate a therapeutic effect of miR-335-3p on CNH-established PAH in mice.

### Inhibition of NF-κB activation alleviates CNH-induced PAH

**Figure 4:**
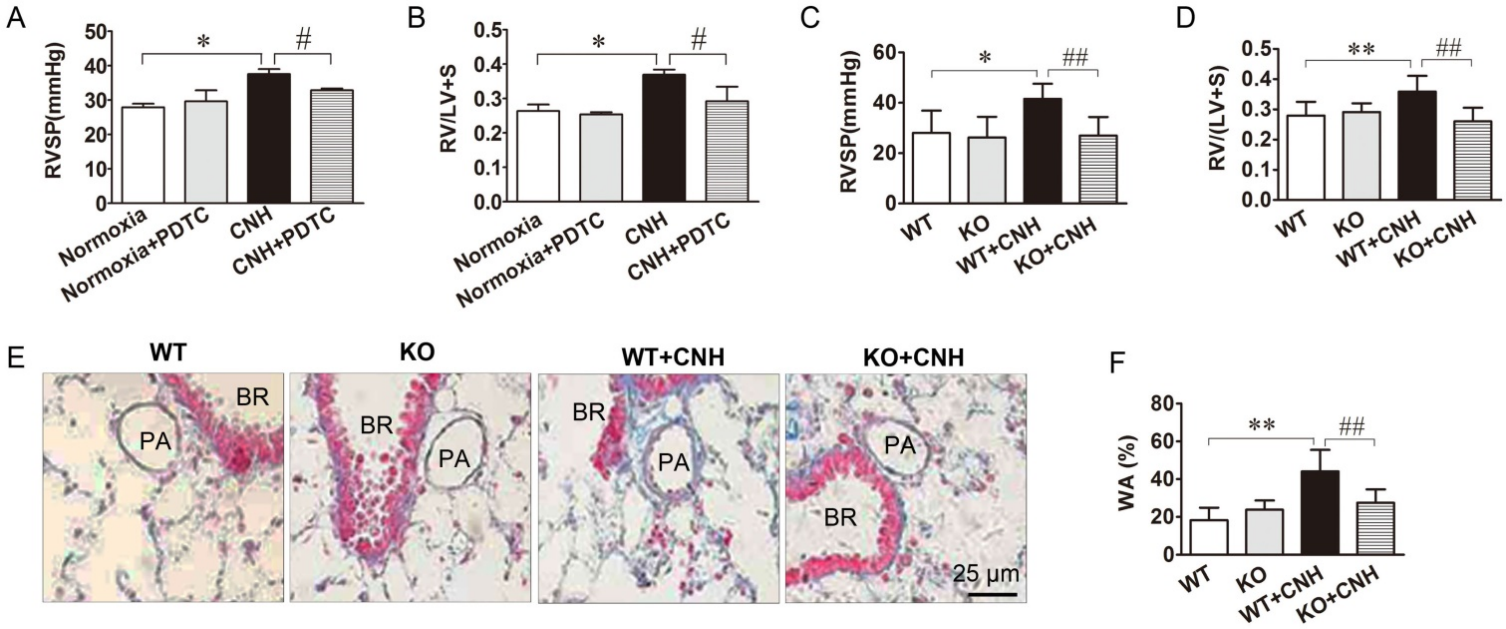
** Blockade of NF-κB ammeliorates CNH-induced PAH and pulmonary vascular remodeling.** PDTC treatment suppressed CNH-induced increase in RVSP **(A)** and RV/(LV+S) **(B)**. CNH exposure significantly increased RVSP **(C)** and RV/(LV+S) ratio **(D)** in WT but not in KO mice. **(E)** Representative images of the pulmonary vascular remodeling in lung sections subjected to Masson trichrome staining in WT and KO mice. **(F)** Quantitative analysis of pulmonary arterial wall area/total cross area (WA/TA). Values shown are means±s.e.m. N=5-8 per group. *p<0.05, **p<0.01 *vs.* Normoxia. #*p*<0.05, ##*p*<0.01 *vs.* CNH. PA, pulmonary artery. BR, bronchiole.

To test the role of NF-κB in a mouse CNH-induced PAH model, we treated mice with PDTC, an inhibitor of NF-κB, and examined the ensuing effects on CNH-induced PAH. Two-way ANOVA indicated a significant effect of CNH (F_(1,15)_=13.678; *p*=0.003) and CNH×PDTC (F_(1,15)_=5.620; *p*=0.035) on RVSP, but no significant effect of PDTC alone (F_(1,15)_=1.729; *p*=0.213). An analogous effect of CNH (F_(1,26)_=19.325; *p*=0.0006) and CNH×PDTC (F_(1,26)_=4.703; *p*=0.041) but not PDTC alone (F_(1,26)_=0.371; *p*=0.549) was observed also in relation to the RV/(LV+S) ratio. Post hoc analysis revealed that CNH exposure significantly increased RVSP and the RV/(LV+S) ratio relative to normoxia control mice. Treatment of CNH-exposed mice with PDTC diminished CNH-induced increases in RVSP (Figure 4A) and the RV/(LV+S) ratio (Figure 4B), indicating a preventive effect of NF-κB on CNH-induced PAH in mice.

To further confirm whether NF-κB was involved in CNH-induced PAH in mice, we assessed the effect of CNH on PAH development in p50^-/-^ mice. Two-way ANOVA supported a significant effect of CNH (F_(1,15)_=5.355; *p*=0.039), NF-κB (F_(1,15)_=6.670; *p*=0.024), and CNH×NF-κB (F_(1,15)_=4.451; *p*=0.05) on RVSP. Similarly, CNH (F_(1,15)_=5.031; *p*=0.04), NF-κB (F_(1,15)_=10.369; *p*=0.006), and CNH×NF-κB (F_(1,15)_=14.622; *p*=0.002) exerted a significant effect on the RV/(LV+S) ratio. Post hoc analysis showed that wild-type (WT) mice exposed to CNH for 5 weeks exhibited a significant increase in RVSP (Figure 4C) and the RV/(LV+S) ratio (Figure 4D) compared with mice in the normoxia control group. In contrast, the same elevated CNH-induced parameters were significantly diminished in p50^-/-^ mice. These findings directly confirm that NF-κB-deficient mice are resistant to CNH-induced PAH.

We next evaluated the effect of CNH on pulmonary artery remodeling using Masson trichrome staining in p50^-/-^ mice treated with CNH or normoxia controls. WT mice exposed to CNH for 5 weeks exhibited a marked increase in the wall thickness of pulmonary small distal arteries when compared with normoxic mice. This same CNH-induced increase in wall thickness was significantly diminished in p50^-/-^ mice (Figure 4E, Figure 4F). There was no difference between WT and p50^-/-^ mice under normoxia. These findings clearly suggest the involvement of NF-κB in CNH-induced pulmonary vascular remodeling in mice.

### MiR-335-3p is transcriptionally regulated by NF-κB

**Figure 5:**
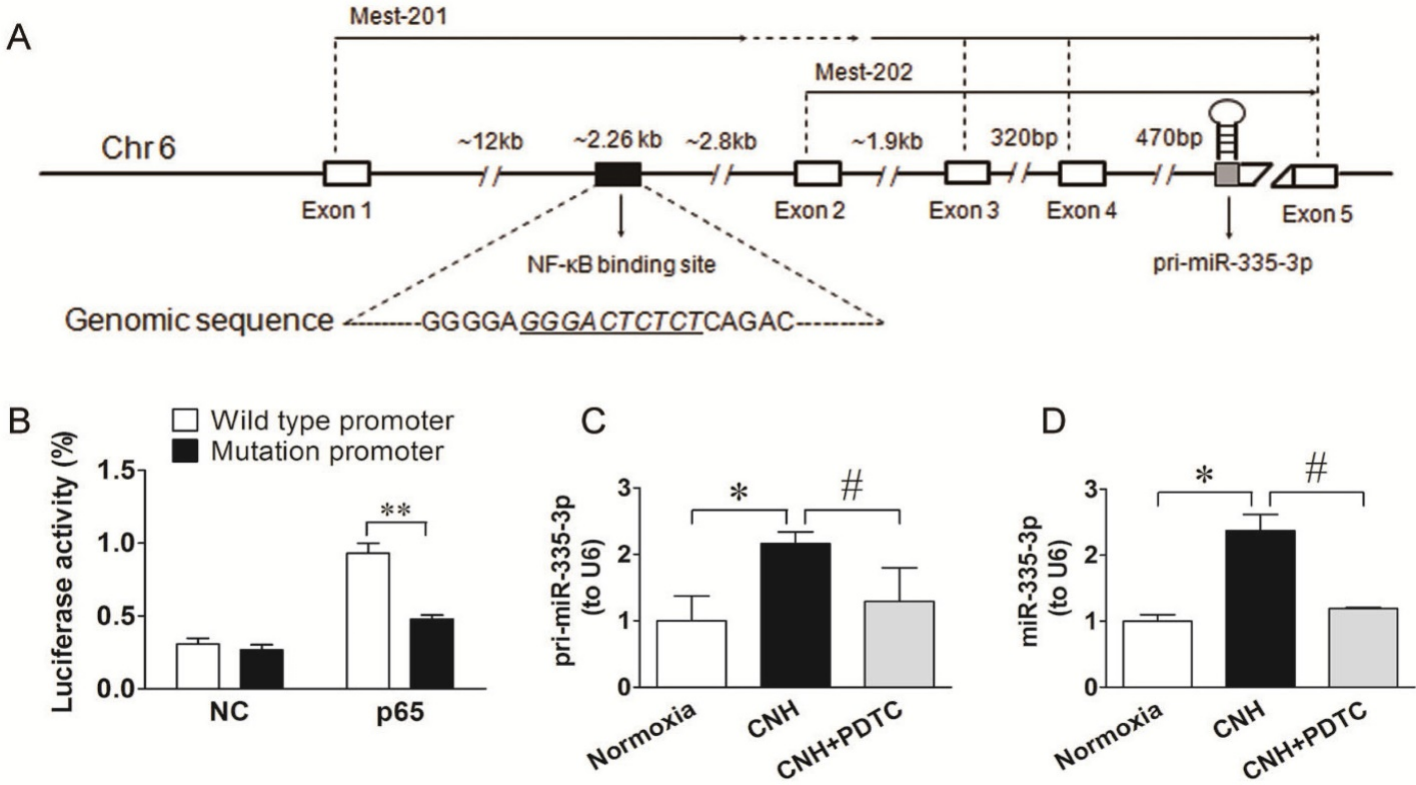
** NF-κB is a transcriptional regulator upstream of miR-335-3p**. **(A)** Schematic representation of putative NF-κB binding site in the promoter region of miR-335-3p, and the corresponding mutation was generated within the binding site. **(B)** Luciferase assay showed that the activity of the luciferase reporter fusing wild-type promoter, but not the mutated promoter with mutation within NF-κB binding site, was increased significantly after p65 cDNA treatment. **p<0.01. NC refers to an empty vector. **(C)** qRT-PCR analysis of pri-miR-335-3p and miR-335-3p **(D)** expression in the lungs of mice. Values shown are means±s.e.m. N=5-8 per group. *p<0.05 *vs.* Normoxia. #*p*<0.05 *vs.* CNH.

Given that NF-κB could transcriptionally regulate downstream target miRs by binding directly to their promoters 37, 38, and that activated NF-κB signaling contributed to CNH-induced PAH in mice, we hypothesized that CNH-induced upregulation of miR-335-3p might be NF-κB-related. Therefore, first, we performed a database search (http://asia.ensembl.org/index.html) to identify the miR-335-3p promoter. We found that the mouse miR-335-3p gene was located on chromosome 6 and consisted of five exons separated by ~17 kb of genomic sequence. A putative NF-κB binding site (*GGGACTCTCT*) was found ~12 kb upstream of exon 1, which was localized furthest upstream from the transcription start site (Figure 5A). To confirm the role of NF-κB signaling in the transcriptional regulation of miR-335-3p, we constructed a WT luciferase reporter vector containing 2260 bp of the mouse pri-miR-335-3p 5′ proximal genomic region and a mutant luciferase reporter vector, in which the NF-κB binding sites were deleted. HEK293 cells were co-transfected with the luciferase reporter vector and p65 cDNA vector. Activation of NF-κB expression increased luciferase activity driven by the WT miR-335-3p promoter, whereas p65 cDNA had no significant effect on the activity of mutant miR-335-3p promoter (Figure 5B). To further investigate the role of NF-κB in activating miR-335-3p transcription in the CNH-induced PAH mouse model *in vivo*, we performed qRT-PCR analysis of pri-miR-335-3p and miR-335-3p expression in CNH mouse lungs treated with the NF-κB inhibitor PDTC. Both pri-miR-335-3p and miR-335-3p expression were significantly increased after CNH-treatment; however, this effect was significantly suppressed by PDTC pretreatment (Figure 5C, Figure 5D). Together, these results suggest that CNH-induced miR-335-3p upregulation depends on NF-κB binding to the primary miR-335-3p promoter.

### MiR-335-3p downregulates apelin-APJ signaling in mouse lungs

**Figure 6:**
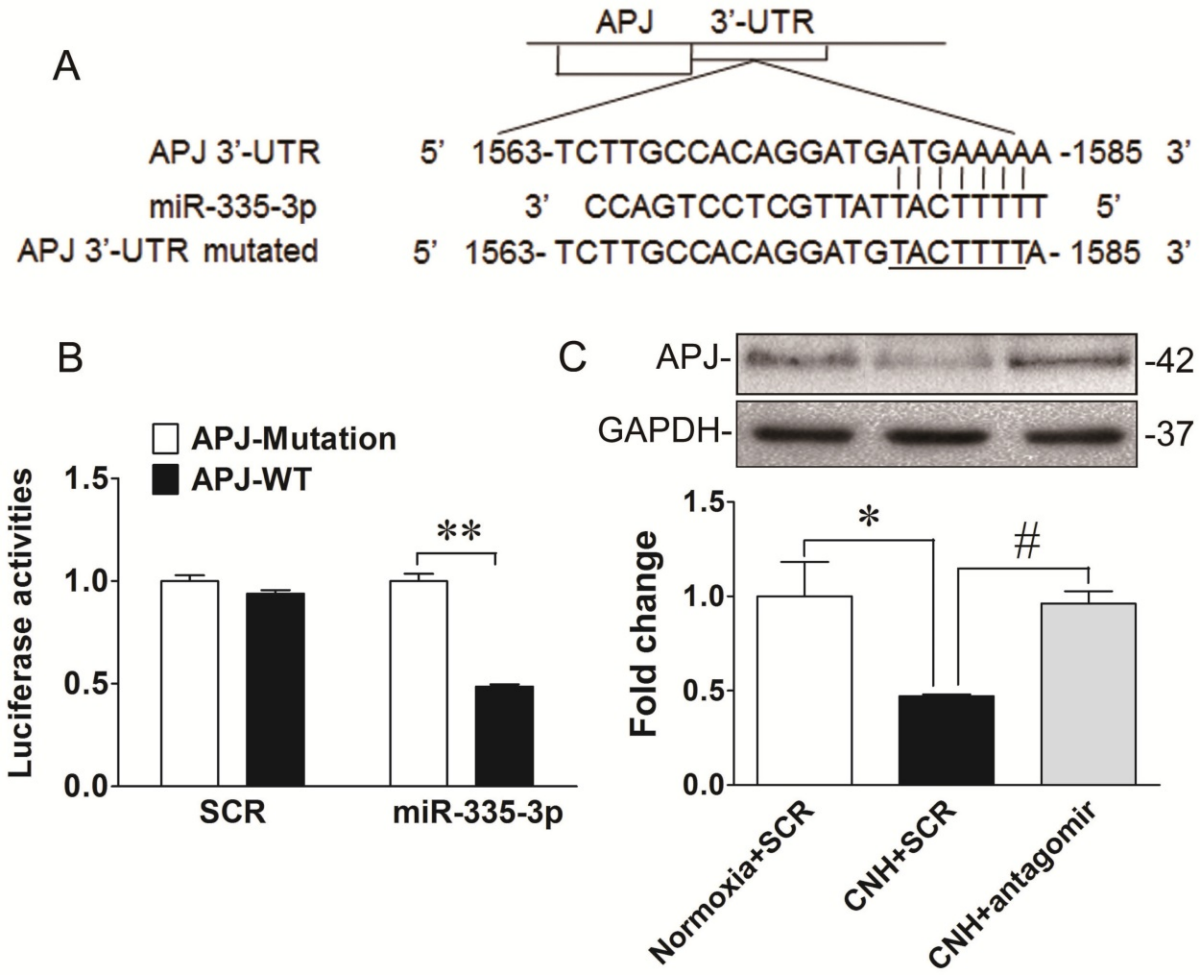
** APJ is a direct downstream target of miR-335-3p. (A)** Diagram of the miR-335-3p binding sites in the 3′-UTR of the APJ gene. **(B)** Luciferase reporter gene assay 3′-UTR of APJ with target and its mutant along with miR-335-3p agomir or scramble control vectors in HEK293 cells. **p<0.01.** (C)** Western blotting analysis of APJ expression in the lungs of mice. **(D)** Quantification of APJ expression bands in **(C)** was done by densitometry and normalized to GAPDH. Values shown are means±s.e.m. N=5-8 per group. *p<0.05 *vs.* Normoxia. #*p*<0.05 *vs.* CNH.

MiRs mediate post-transcriptional regulation by binding directly to sequences analogous to their seed region in the 3′-UTR of target mRNAs. To identify the downstream molecular candidates of miR-335-3p, we examined the predicted miR-335-3p targets using bioinformatics tools. Using an available prediction algorithms (Targetscan), we produced a comprehensive list of all possible miR-335-3p targets. We honed onto APJ as a potential target of miR-335-3p because it contained a conserved miR-335-3p seed sequence in its 3′-UTR (Figure 6A). Importantly, apelin and APJ have been consistently demonstrated to be critical players in PAH and were reported to localize to PASMCs 39-42. These data led us to speculate that apelin/APJ might be functional downstream targets of miR-335-3p during CNH-induced PAH. To verify this hypothesis, we first transfected HEK293 cells with a plasmid containing a luciferase-APJ 3′-UTR construct and found co-transfection with miR-335-3p agomir decreased luciferase activity. This finding directly confirmed the binding of miR-335-3p to the 3′-UTR of APJ and a consequent reduction in its transcription (Figure 6B). We then performed western blotting to test the level of APJ in CNH-treated mouse lungs. As shown in Figure 6C and Figure 6D, APJ expression was reduced in CNH-treated mouse lungs compared with normoxia controls and, importantly, this decrease was prevented by miR-335-3p antagomir treatment. The data presented thus far provide evidence that increased levels of miR-335-3p might contribute to the pathogenesis of hypoxic PAH through repression of key cellular targets such as APJ. Taken together, our data suggest that exacerbated expression of miR-335-3p during CNH-induced PAH progression, proceeds at least in part through inhibition of the apelin-APJ signaling pathway.

#### Apelin-13 treatment diminishes CNH-induced PAH and ameliorates pulmonary artery remodeling in mouse lungs

**Figure 7:**
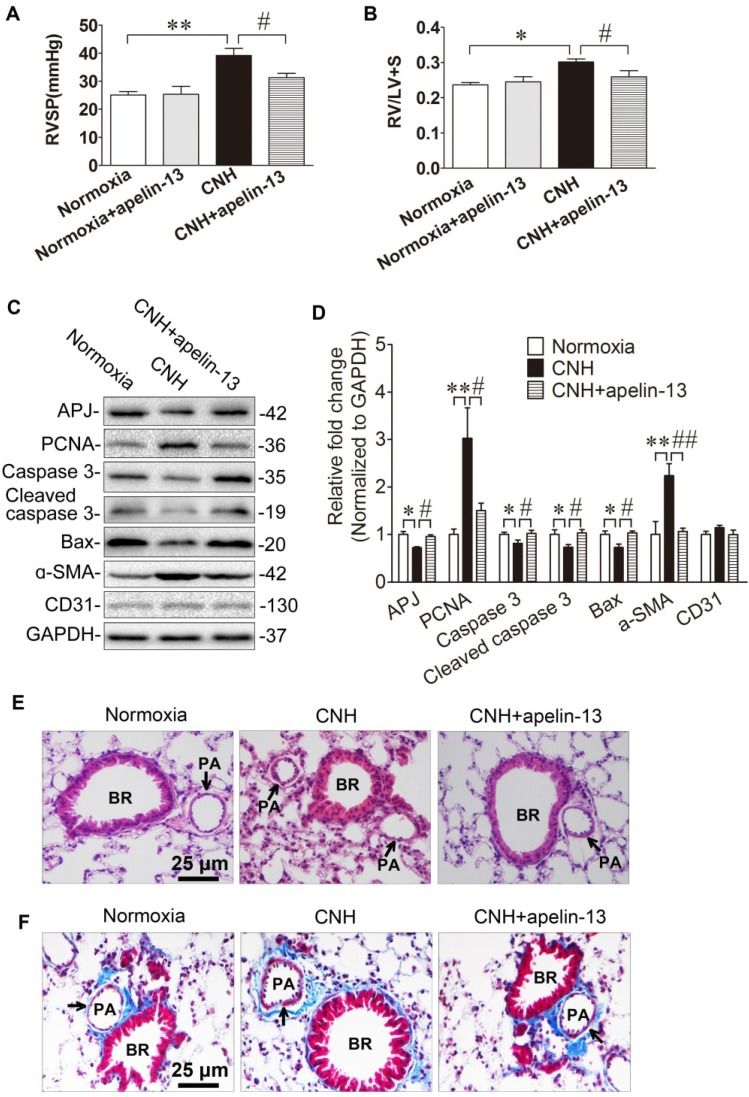
** Amelioration of apelin-13 in CNH-induced PAH and pulmonary vascular remodeling in mice.** Apelin-13 treatment suppressed CNH-induced increase in RVSP **(A)** and RV/LV+S **(B)**. **(C)** Western blotting analysis of relative proteins expression in the lungs of mice. **(D)** Quantification of proteins expression bands in **(C)** was done by densitometry and normalized to GAPDH.** (E)** Representative images of H&E staining of lung sections.** (F)** Representative Masson trichrome staining of lung section. Values shown are means±s.e.m. N=5-8 per group. *p<0.05, **p<0.01 *vs.* Normoxia. #*p*<0.05, ##*p*<0.01 *vs.* CNH. PA, pulmonary artery. BR, bronchiole.

APJ and its cognate endogenous ligand apelin are important for the development of PAH, and disruption of the apelin-APJ axis plays a major part in the pathogenesis of PAH 39-41. To firmly establish the mechanistic role of miR-335-3p given its impact on APJ expression, we asked whether activation of APJ *in vivo* could attenuate CNH-induced PAH. Two-way ANOVA indicated a significant effect of CNH (F_(1,25)_=11.005; *p*=0.004) and CNH×apelin (F_(1,25)_=5.239; *p*=0.036) on RVSP, while no such effect was observed for apelin alone (F_(1,25)_=0.122; *p*=0.732). The same effect of CNH (F_(1,25)_=11.213; *p*=0.003) and CNH×apelin (F_(1,25)_=4.595; *p*=0.043) but not apelin alone (F_(1,25)_=1.275; *p*=0.27) was observed also regarding the RV/(LV+S) ratio. Post hoc analysis showed that RVSP in the CNH group, but not in the CNH×apelin-13 group, was significantly higher relative to the normoxia control group (Figure 7A). Consistent with changes in RVSP, the RV/(LV+S) ratio was markedly enhanced in CNH-exposed mice, which was strongly attenuated with apelin-13 treatment compared with CNH-exposed mice treated with vehicle only, (Figure 7B). To determine whether CNH exposure could induce cellular proliferation while apelin-13 administration ameliorated this effect of CNH, we assessed relative protein levels in mouse lungs. We found that PCNA and ɑ-SMA were significantly increased while total/cleaved caspase 3 and Bax were markedly decreased in CNH-treated mice relative to the normoxia control group; however, this effect was alleviated following apelin-13 administration (Figure 7C, Figure 7D). Concomitantly, we noted that apelin-13 treatment significantly suppressed CNH-induced pulmonary artery remodeling (Figure 7E, Figure 7F). Together, these data indicate that amelioration of apelin-13 in CNH-induced PAH in mice occurs through inhibition of cellular proliferation and pulmonary artery remodeling.

## Discussion

PAH is characterized by progressive remodeling of the pulmonary artery, which can result in increased pulmonary vascular resistance, right heart failure, and even death. Growing evidence suggests that miRs, which act as important regulators of gene expression at the post-transcriptional level, play essential roles in PAH but could have also a preventive and therapeutic effect on the disease 14, 17, 18. Our study provides the first evidence demonstrating a novel role of the NF-κB/miR-335-3p/APJ axis in regulating CNH-induced PAH. Indeed, we report increased NF-κB activation and high miR-335-3p expression but decreased APJ expression in lung tissues of a CNH-induced PAH mouse model. Conversely, blocking NF-κB by pharmacological means resulted in a decrease in miR-335-3p but an increase in APJ expression. Similarly, repression of miR-335-3p restored APJ expression and lessened the hypoxic effect on RVSP and RVH. We further identified and confirmed NF-κB as a direct upstream transcriptional regulator of miR-335-3p, and APJ as a downstream target of miR-335-3p. Moreover, exogenous apelin-13 treatment diminished CNH-induced PAH and ameliorated pulmonary artery remodeling. Together, our results suggest that activation of NF-κB leads to an upregulation of miR-335-3p in response to hypoxia, which promotes repression of APJ *in vivo*, drives pulmonary artery remodeling, and induces PAH.

Numerous studies have indicated the involvement of miRs in regulating PAH development, sparking clinically relevant research on the regulation of endogenous miRs in PAH models 10, 13-15, 17, 18, 43. In the present study, we first investigated the potential involvement of miRs in the regulation of CNH-induced PAH by characterizing the profile of miRs expressed in mouse lung tissues following CNH exposure. By combining microarray and qRT-PCR results, we identified miR-335-3p to be robustly upregulated in lung tissues in a CNH-induced PAH mouse model. While previous studies have demonstrated that miR-335 is critically involved in the regulation of reparative activities of human mesenchymal stem cells 44 and promotes mesendodermal lineage segregation 45, none, to the best of our knowledge, has comprehensively defined the mechanistic role of miR-335-3p in PAH pathogenesis. Here, we demonstrate that blocking miR-335-3p could prevent CNH-induced PAH development and attenuate pulmonary artery remodeling. Emerging evidence suggests that the expression of miRs can be regulated at transcriptional levels 46, and NF-κB has been shown to promote miR-223-3p transcriptional induction, thus facilitating the proliferation and migration of gastric cancer cells 47. Increased NF-κB activation was previously observed in mouse and rat lungs following hypoxia 33 or monocrotaline 27, 29 stimulation. In this study, promoter analysis led to the identification of a putative NF-κB binding site located in the promoter region of the *miR-335-3p* gene. Luciferase reporter assays clearly indicated that CNH-induced elevation of miR-335-3p expression occurred in an NF-κB-dependent manner. In addition, by using both pharmacological and genetic approaches, we demonstrated that NF-κB was involved in CNH-induced PAH because NF-κB inhibitor treatment ameliorated CNH-induced PAH in mice, whereas NF-κB knockout mice were resistant to CNH-induced PAH. Our results are in agreement with previous reports that NF-κB activation is involved in PAH development 27-29, 33.

The post-transcriptional regulation of gene expression by miRs depends on them binding to either specific sites in the 3′-UTR or the coding regions of their target mRNAs, resulting in degradation or translational stalling 7. Bioinformatics miR target prediction yielded several potential targets, but the *APJ* gene had particularly high scores. APJ is a validated target of miR-335-3p and, together with its ligand apelin, is highly expressed in the pulmonary vasculature 48. Both are also known to be potent regulators during PAH development 39, 41, 42, 49, 50. Mice with apelin deletion develop a more severe form of PAH when exposed to chronic hypoxia 40, directly confirming the involvement of the apelin-APJ axis in the pathogenesis of hypoxic PAH. We first verified and confirmed that miR-335-3p directly bound to the complementary sites on the 3′-UTR of APJ and decreased its protein levels. Because miR-335-3p expression was markedly elevated in mouse lung tissues, we then investigated its upregulation by examining the expression of APJ. Delivery of antagomir-335-3p restored APJ expression, which was downregulated by exposure to CNH. Finally, injection of apelin-13 attenuated CNH-induced cellular proliferation and pulmonary arterial remodeling, and ameliorated PAH phenotypes (RVSP, RVH) in mice exposed to CNH. Our data are in line with previous reports 40, 41, 49. Notably, the apelin/APJ combination has been found to retard proliferation of PASMCs during PAH development 51, 52. Pulmonary arterial endothelial cells with apelin deletion were reported to promote PASMCs proliferation; however, proliferation was suppressed and apoptosis was induced when apelin levels were restored. Moreover, apelin administration reversed PAH in mice with reduced production of apelin resulting from deletion of PPARγ in endothelial cells 52. In addition, apelin treatment inhibited the proliferation and migration of PASMCs mediated by the PI3K/Akt/mTOR signaling pathways under hypoxia 51. Together, these findings suggest that miR-335-3p promotes CNH-induced PAH by directly targeting APJ, while APJ exerts a protective role in the development of CNH-induced PAH. Viable conditional KO mice, whose miR-335-3p gene is inactivated in the lungs, would allow further assessment of *miR-335-3p* function during CNH-induced PAH. This would advance our understanding of the exact mechanism by which miR-335-3p contributes to hypoxic PAH. Here, we propose that augmented miR-335-3p activation during CNH exposure plays a crucial role in promoting the development of PAH by targeting APJ. However, we cannot rule out the possibility that miR-335-3p exhibits its biological role by targeting other downstream genes closely associated with PAH. Additional studies are required to confirm this possibility.

**Figure 8:**
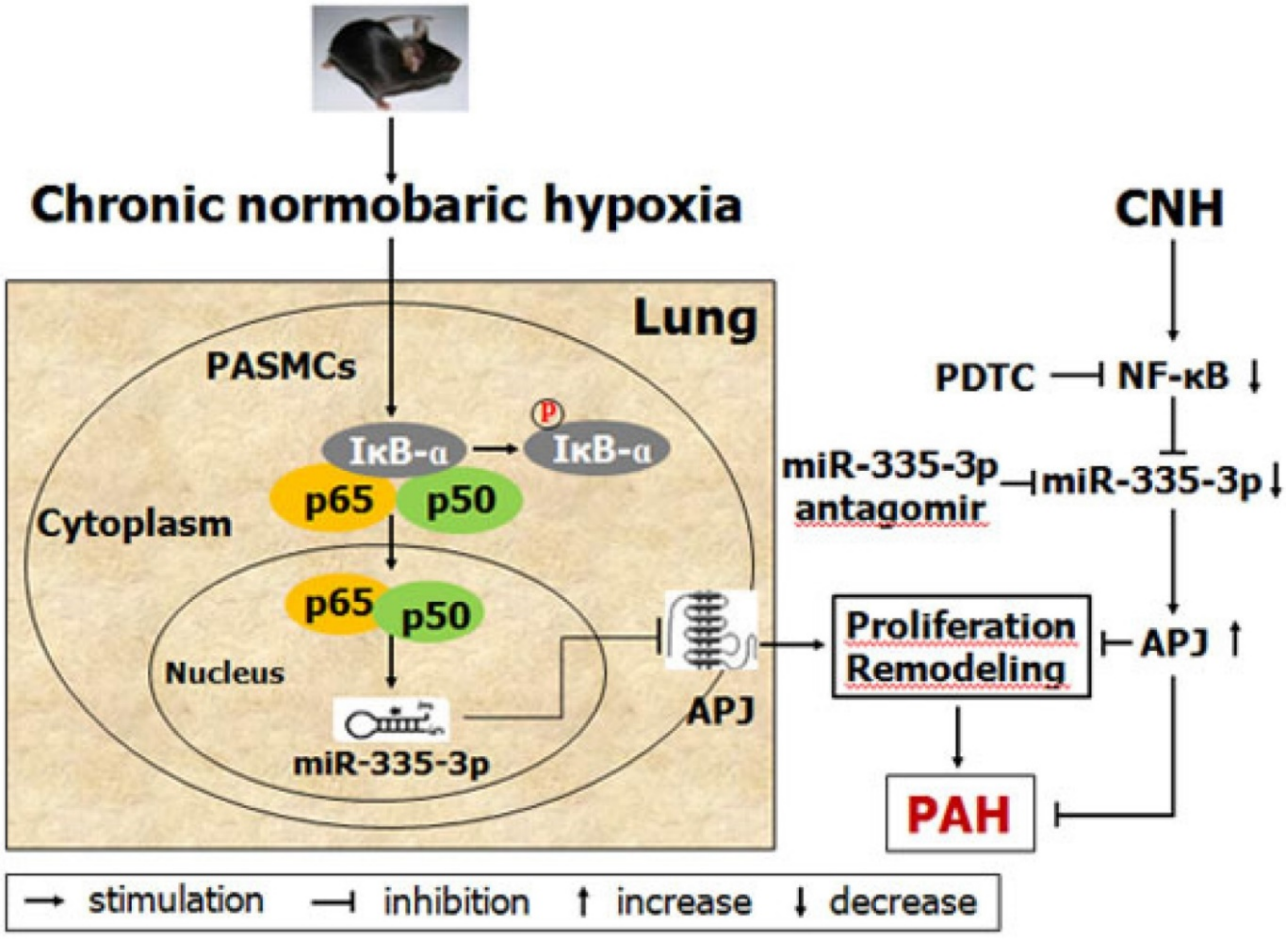
Schematic model showing the role and mechanism of miR-335-3p in chronic normobaric hypoxia (CNH)-induced pulmonary arterial hypertension (PAH) in mice.

In summary, this study provides the first evidence that NF-κB-mediated transcriptional upregulation of miR-335-3p expression is positively involved in the progression of CNH-induced PAH, and that miR-335-3p modulates the development of CNH-induced PAH *via* inhibition of APJ (Figure 8). Our data highlight the critical role of the NF-κB/miR-335-3p/APJ axis in CNH-induced PAH; consequently, miR-335-3p may be a potential therapeutic target against PAH during hypoxia.

## Supplementary Material

Supplementary figure S1.

## Abbreviations

3′-UTR: 3′-untranslated region
CNH: chronic normobaric hypoxia
miR: microRNA
NF-κB: nuclear factor-kappa beta
PAH: pulmonary arterial hypertension
PASMCs: pulmonary arterial smooth muscle cells
PDTC: pyrrolidine dithiocarbamate
RVH: right ventricular hypertrophy
RVSP: right ventricular systolic pressure
RV/(LV+S): right ventricle to left ventricle plus septum
WT: wild-type
