# Supplementary material for: Upregulation of miR-335-3p by NF-κB Transcriptional Regulation Contributes to the Induction of Pulmonary Arterial Hypertension via APJ during Hypoxia
Source: Int J Biol Sci. 2020 Jan 1;16(3):515–28. doi: 10.7150/ijbs.34517 (PMC6990898; doi:10.7150/ijbs.34517)
Supplement: Supplementary file 1 — Supplementary figure S1. [file ijbsv16p0515s1.pdf]

Supplementary Figure S1

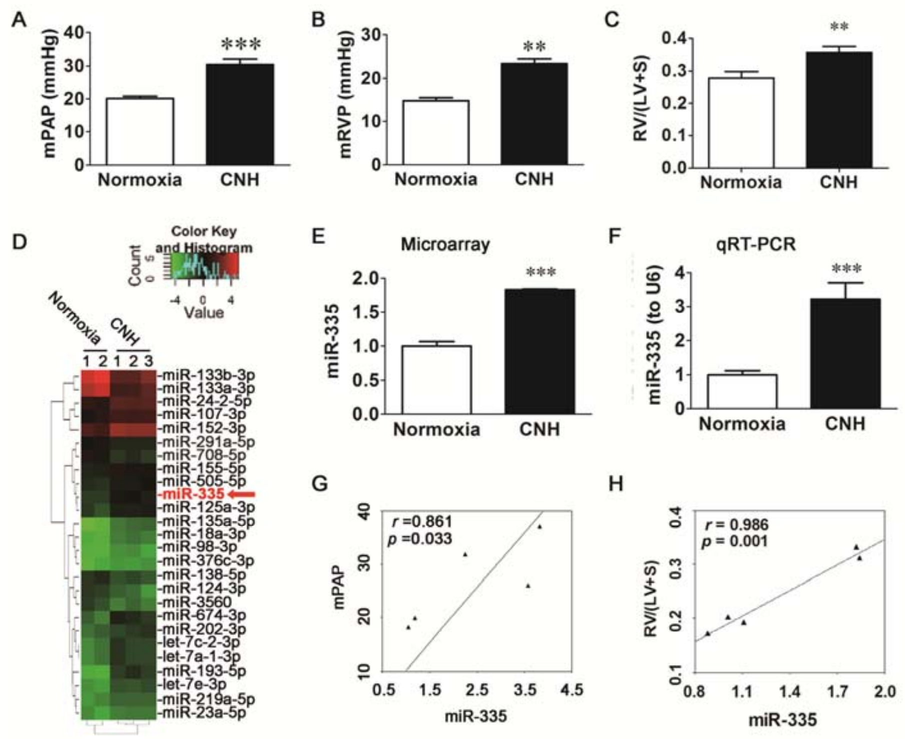

Figure S1: Chronic normobaric hypoxia (CNH) exposure induces pulmonary arterial hypertension (PAH) and upregulates miR-335 expression in the lungs of rat. CNH exposure significantly increased mPAP **(A)**, mean right ventricular pressure (mRVP) **(B)**, and RV/LV+S **(C)** in rats (N=5-8 per group). **(D)** Heat map and sample clustering analysis of the differential expressed miRs in the lungs of rat. **(E)** Microarray and quantitative RT-PCR **(F)** analysis of miR-335 expression in the lungs of rat (N=2-3 per group). **(G)** Correlation analysis between miR-335 and mPAP, and **(H)** Correlation analysis between miR-335 and RV/LV+S. Values shown are means±s.e.m. ***p*<0.01, ****p*<0.001 *vs.* Normoxia.
